# Strength and timing of motor responses mediated by rebound firing in the cerebellar nuclei after Purkinje cell activation

**DOI:** 10.3389/fncir.2013.00133

**Published:** 2013-08-21

**Authors:** Laurens Witter, Cathrin B. Canto, Tycho M. Hoogland, Jornt R. de Gruijl, Chris I. De Zeeuw

**Affiliations:** ^1^Netherlands Institute for Neuroscience, Royal Netherlands Academy of Arts and SciencesAmsterdam, Netherlands; ^2^Department of Neuroscience, Erasmus Medical CenterRotterdam, Netherlands

**Keywords:** olivo-cerebellar network, Purkinje cells, rebound, cerebellar nuclei, motor control

## Abstract

The cerebellum refines the accuracy and timing of motor performance. How it encodes information to perform these functions is a major topic of interest. We performed whole cell and extracellular recordings of Purkinje cells (PCs) and cerebellar nuclei neurons (CNs) *in vivo*, while activating PCs with light in transgenic mice. We show for the first time that graded activation of PCs translates into proportional CN inhibition and induces rebound activity in CNs, which is followed by graded motor contractions timed to the cessation of the stimulus. Moreover, activation of PC ensembles led to disinhibition of climbing fiber activity, which coincided with rebound activity in CNs. Our data indicate that cessation of concerted activity in ensembles of PCs can regulate both timing and strength of movements via control of rebound activity in CNs.

## Introduction

The cerebellum integrates sensory and motor information to learn and refine the timing of motor performance. Sensory and motor information enters the cerebellar cortex via climbing fibers that originate in the inferior olive (IO) and via mossy fibers that originate in a variety of precerebellar sources (Ito, 1984). Climbing fibers synapse onto Purkinje cells (PCs) in rostrocaudally oriented cerebellar cortical zones (Ozden et al., 2009) and generate complex spikes (CSs) (Eccles et al., 1964). There is a one-to-one relation between IO neuron firing and the occurrence of a CS in the target PC (Eccles et al., 1966). Apart from CSs, which occur at a relatively low rate of about 1 Hz at rest and up to 5–8 Hz during optimal stimulation (Llinas and Volkind, 1973; Llinas and Yarom, 1986; Llinas and Sasaki, 1989; Sasaki et al., 1989; Lang et al., 1999), PCs fire simple spikes (SSs) at 50–100 Hz (Latham and Paul, 1971). SSs are intrinsically driven in PC cell bodies by resurgent sodium currents (Raman and Bean, 1997; Afshari et al., 2004; Aman and Raman, 2007) and are modulated by excitatory and inhibitory inputs from the mossy fiber—parallel fiber pathway and molecular layer interneurons (MLIs), respectively (Jacobson et al., 2008; Oldfield et al., 2010). The activity of MLIs, the axons of which target PCs within an individual sagittal zone, can be influenced by both parallel fibers and climbing fibers (Ekerot and Jorntell, 2001; Jorntell and Ekerot, 2002, 2003; Szapiro and Barbour, 2007; Bosman et al., 2010; Mathews et al., 2012; Badura et al., 2013). Ultimately, information from the zones of PCs is processed by cerebellar nuclei neurons (CNs) (Palay and Chan-Palay, 1974; Palkovits et al., 1977), which can inhibit the IO (De Zeeuw et al., 1988; Angaut and Sotelo, 1989; Ruigrok and Voogd, 1990; Fredette and Mugnaini, 1991) or provide an excitatory projection to a variety of premotor targets in the brainstem or thalamus (Bentivoglio and Kuypers, 1982; Voogd and Ruigrok, 1997; Garwicz, 2000).

Given the central hub position of the PC—CN projection, it is key to understand how PCs and CNs encode their information and how their activities integrate to control motor behavior (Aizenman and Linden, 1999; Alvina et al., 2008; Hoebeek et al., 2010; Bengtsson et al., 2011; De Zeeuw et al., 2011; Witter et al., 2011a; Person and Raman, 2012a,b). One of the main questions is to what extent behaviorally relevant information is transferred by individual PCs through rate coding or by synchronously timed activity and silent periods in ensembles of PCs (Bell and Grimm, 1969; Sjolund et al., 1977; Sasaki et al., 1989; Welsh et al., 1995; Levin et al., 2006; Walter et al., 2006; Heck et al., 2007; Catz et al., 2008; de Solages et al., 2008; Ozden et al., 2009; Schultz et al., 2009; Wise et al., 2010; Person and Raman, 2012a,b). Since PC axons are, like climbing fibers, organized in sagittal zones enabling ensembles of PCs to innervate a specific set of CNs, it is conceivable that PCs employ this modular organization to direct CN activity. A potential mechanistic target for such modulation is rebound activity in CN neurons, which is characterized by an elevated firing frequency following release from PC inhibition and which may rely on concerted activation and/or silencing of PCs (Llinas and Muhlethaler, 1988; Aizenman and Linden, 1999; Molineux et al., 2006, 2008; Tadayonnejad et al., 2010; Engbers et al., 2011). Rebound activity could impact postsynaptic structures such as the thalamus, red nucleus, IO and lateral reticular formation (Teune et al., 2000), and eventually motor behavior (De Zeeuw et al., 2011). However, whether CN rebound firing can be proportionally induced by graded and timed modulation of activity in specific ensembles of PCs *in vivo* and whether such a titrating process can shape motor output accordingly has not been resolved. Investigating these questions has been hampered by the difficulty of classical electrophysiological tools to stimulate specific cell types selectively, let alone to stimulate these cells in small ensembles, and to record from CNs in the whole cell mode *in vivo*. Here we used a genetic approach to express the H134R variant of channelrhodopsin-2 (ChR2) specifically in PCs by crossing L7-cre (Oberdick et al., 1990) with ChR2(H134R) (Ai32line) mice (Madisen et al., 2012). We performed whole cell and extracellular recordings of PCs and CNs as well as video recordings of tail and limb movements, while stimulating ensembles of PCs with different intensities of light during precisely determined, yet variable time periods. We found that graded activation and subsequent cessation of sagittal PC ensembles *in vivo* translated into corresponding CN inhibitions and rebounds, which in turn evoked proportional muscle contractions and movements, indicating that rebound firing may orchestrate activity in premotor brain areas and thereby control muscle activity.

## Results

**Figure 1:**
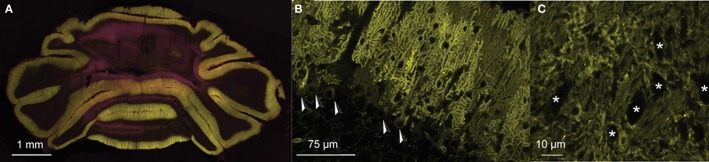
**PC-specific expression of ChR2(H134R)-eYFP under control of L7-pcp2. (A)** Coronal section of the cerebellum of an L7-ChR2(H134R)-eYFP mice. PC and molecular layers show dense expression of the ChR2-eYFP fusion protein (eYFP: yellow). **(B)** Detail of a sagittal section of an L7-ChR2(H134R)-eYFP mouse. ChR2-eYFP protein expression was restricted to PC membranes. PC somata are indicated with arrowheads. Note that MLIs are visible as small dark exclusions in the PC arborizations of the molecular layer. The neuronal expression of ChR2-eYFP fusion protein was found only in PCs of the cerebellum, but not in other neuronal structures in the rest of the brain. **(C)** Detail of PC axons innervating the cerebellar nuclei. CNs are marked with ‘*’. Counterstain in **(A)** with DAPI (pink).

To assess network connectivity between PC ensembles and CNs at the physiological level we performed whole cell and extracellular *in vivo* recordings of PCs and CNs in genetically modified mice that expressed ChR2(H134R)-eYFP under the L7 promotor (Oberdick et al., 1990; Madisen et al., 2012). Expression of the channelrhodopsin-2/eYFP fusion protein was restricted exclusively to PCs in these mice (Figures 1A–C). In the cerebellar nuclei, the fusion protein was present in axons and PC terminals surrounding CNs. There was no expression in other neuronal cell types in the cerebellum or the rest of the brain.

### Light-driven purkinje cell modulation

**Figure 2:**
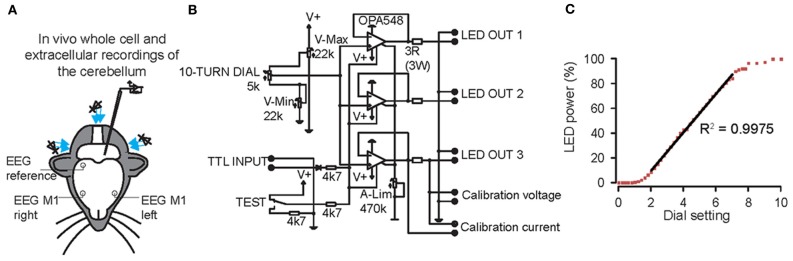
**Graded whole field light stimulation by three blue light emitting diodes (LEDs) positioned around the cerebellum of mice controlled by a custom-built linear LED driver. (A)** Overview of the experimental set-up. Whole cell and extracellular recordings were made from Purkinje cells (PCs), cerebellar nuclei neurons (CNs), molecular layer interneurons (MLIs) and granule cells. At the same time, bilateral electroencephalogram (EEG) was recorded from motor cortex, referenced on the right parietal cortex. **(B)** Circuit diagram of one channel of the LED driver. A 10-turn dial permitted setting of light-intensity during experiments. A TTL input can be used to trigger the light from an external source. V-Max, V-Min, and A-Lim (measuring from calibration voltage or current) are used to limit the voltage and current through the LED and to calibrate the 10-turn dial. Up to three LEDs can be connected in parallel on a single channel. **(C)** LED power is a linear function of the dial setting in the range between 20 and 75%.

**Figure 3:**
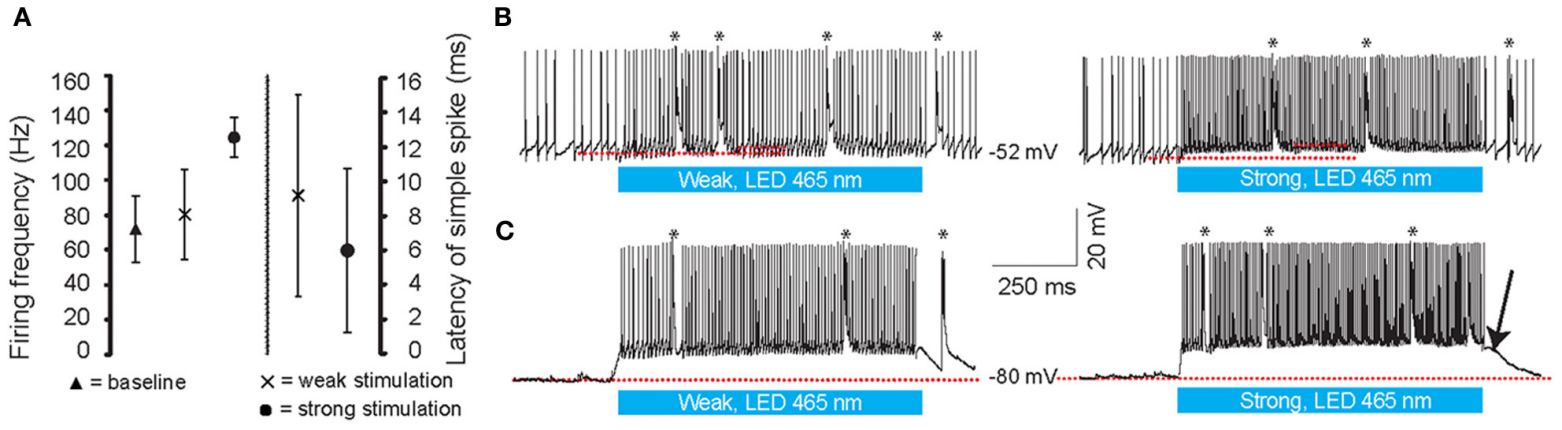
**Whole cell *in vivo* recordings of PCs during optogenetic stimulation. (A)** Latency to the first simple spike (SS) and the increase in firing frequency during the light stimuli at different intensities. **(B,C)** Light stimulation (465 nm, 1000 ms, denoted by the blue bars below the traces) of the cerebellum at weak (left panels) and strong (right panels) light intensities. Both in the upstate **(B)** and downstate **(C)** PCs show graded increases of SSs and CSs during stimulation. Note, ChR2 (H134R) has slow kinetics; at light-offset, the cell remains depolarized for a few milliseconds before it settles back down to a baseline state (arrow). Red lines indicate the subthreshold membrane potentials before and during the light stimulation. Asterisks indicate CSs.

We first made whole cell current clamp and extracellular recordings from PCs *in vivo* in response to light stimulation by three blue LED lights positioned around the cerebellum of anesthetized mice (*N* = 7) (Figure 2A). The LEDs were controlled by a custom-made linear LED driver (Figure 2B), which allowed us to adjust the strength of the light in a linear fashion (see Figure 2C for power curve). PCs were identified by CS and SS activity and the characteristic climbing fiber pause (De Zeeuw et al., 2011). Baseline SS activity (i.e., without light stimulus) was 72 ± 19 Hz (Figure 3A). Enhancing the light from 10 to 100% significantly increased the SS firing frequency of PCs from 80 ± 25 Hz to 124 ± 11 Hz [cell-wise comparison: *t*_(5)_ = −4.742, *p* = 0.005], while it reduced the latency of the first SS from 9.1 ± 5.8 ms to 6.0 ± 4.7 ms [all latencies: *t*_(148)_ = 5.181, *p* < 0.001] (Figures 3A–C). Interestingly, light stimulation was also effective in increasing SS activity when the PC was in the downstate (compare Figures 3B,C) (Loewenstein et al., 2005; Schonewille et al., 2006; Jacobson et al., 2008). We were unable to find a direct response within the first 50 ms of light stimulation in any other cell type in the cerebellar cortex. These data demonstrate that with our stimulus device and protocol we were able to selectively activate PCs in L7-ChR2 (H134R) mice in a reliable and graded manner.

### Graded purkinje cell activation translates into proportional cerebellar nuclei inhibition

**Figure 4:**
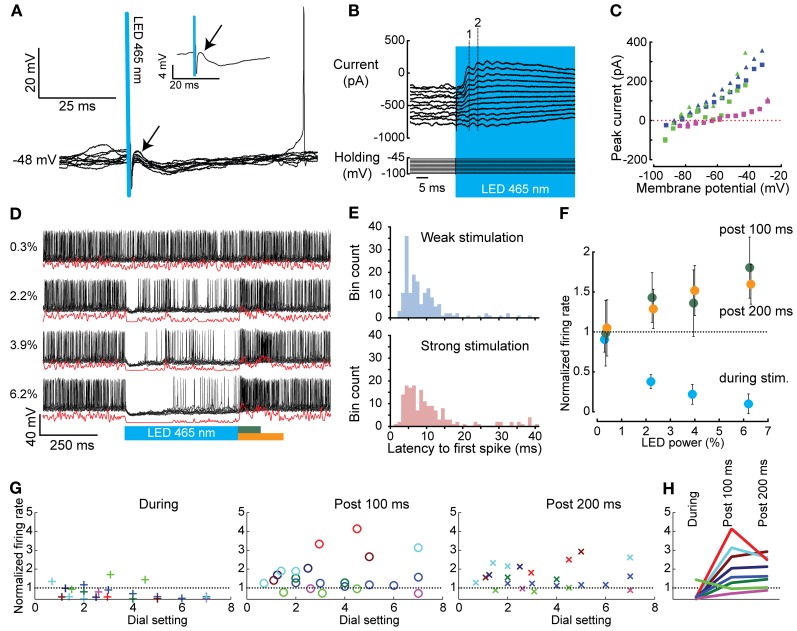
**Whole cell and extracellular *in vivo* recordings of CNs before, during, and after optogenetic activation of PCs. (A)** Brief 1 ms activation of PCs (blue bar) was sufficient to evoke IPSPs in CNs (onset is indicated with an arrow). The inset shows the average trace. **(B)** CN responses (top panel) at various holding potentials (lower panel) used to determine the reversal potential of the evoked events in CNs (stimulus in blue). The dashed numbered lines indicate the peaks of the first (1) and second (2) induced postsynaptic event, respectively. **(C)** IV curves of all CN neurons tested (individual CN neurons are indicated with different colors, squares indicate responses to the first peak, triangles to the second). The reversal potential (E_rev_ = −76.42 ± 8.66 mV) is in agreement with a GABA_*A*_-mediated current. **(D)** Graded PC stimulation (blue bar) [ranging from 0.3 to 6.2 dial setting (Figure 2C)] evoked a graded response in CNs. Black lines indicate raw data, red lines Gaussian convolved traces. **(E)** Histograms of the latency of the first spike in CNs after light-driven PC mediated inhibition. The distribution shows a long tail for both weak and strong stimulation intensities. **(F)** The normalized firing rate (firing rate during stimulation or rebound divided by the prestimulus firing rate) of the CN shown in **(D)** during light stimulus (blue), 100 ms post light stimulus (green dots), or 200 ms post light stimulus (orange dots). **(G)** Summary as in **(F)**, but for all cells. Different neurons are color coded over the three panels. **(H)** The maximal stimulation intensities from the panels in **(F)** are plotted to show that inhibition during the stimulation is necessary for rebound to occur.

We assessed the effect of graded, transient light-driven activation of PCs on CN spiking using different stimulus intensities and frequencies in anesthetized mice. CNs were identified based on their depth measured from the pial surface (1500–2400 μm), their direct response to PC stimulation and their basic electrophysiological properties (Uusisaari et al., 2007; Bengtsson et al., 2011; Witter et al., 2011b). Recordings of CNs were targeted at the interposed nucleus of the cerebellum. CN membrane resistance, capacitance, and firing frequency varied from 11.7–779.1 MΩ, 60.1–772.6 pF, and 0–138.4 Hz, respectively (*N* = 21). Despite the large differences in cell physiological parameters, we were not able to distinguish separate clusters of cells indicative of neuronal subtypes. Also, depth of the recording was not associated with any cell physiological parameter or with the occurrence of rebound firing in these CNs. In current clamp, short light activations (1–3 ms) evoked single inhibitory postsynaptic potentials (IPSPs) in CNs (*N* = 8) (Figure 4A). As expected due to differences in connectivity with PCs and differences in stimulation intensities, these IPSPs varied in amplitude among cells (−3.84 ± 2.13 mV) and onset latency (4.21 ± 1.44 ms). Nevertheless, weaker light activation consistently induced smaller IPSP amplitudes compared to those following strong stimulations in all CNs tested. Next, in voltage clamp we held cells at potentials between −30 and −100 mV while stimulating PCs to calculate the current-voltage relationship (IV curve) of PC input. When stimulating PCs for several tens of ms, summations of postsynaptic currents were indicative of synchronized inputs to CNs (Figure 4B). In most traces we were able to identify two or three summated postsynaptic currents before the inputs became less synchronized. The onset of the evoked currents occurred at 4.04 ± 1.34 ms following the stimulus, while the timing of the first and that of the second peak synaptic current after the onset of the stimulus were 7.08 ± 1.80 ms and 11.93 ± 3.38 ms, respectively (Figure 4B). We determined the reversal potential for the synaptic current from the peaks of both the first and second peak-current. An inward current was observed at strongly hyperpolarized potentials, while outward currents were observed at more depolarized potentials (E_rev_ = −76.42 ± 8.66 mV, slope: 4.37 ± 2.06 pA/mV; *N* = 3) (Figure 4C), which is in line with previously reported characteristics of the PC to CN synapse (Llinas and Muhlethaler, 1988; Zheng and Raman, 2009; Hoebeek et al., 2010). In current clamp mode recordings, we were able to inhibit CNs in a graded fashion using different intensities of light showing that a gradually changing rate of PC firing can lead to a proportional change in CN firing (Figures 4D,F,G). At cessation of the light stimulus, neurons remained inhibited for a variable period depending on the strength of the light stimulus. Following a weak stimulation of 1000 ms the latency to the first spike (time from stimulation offset to first spike) ranged from 1.62 to 448 ms with an average of 41.45 ± 80.85 ms, whereas following a strong stimulation of the same duration it varied from 0.52 to 91.47 ms with an average of 19.37 ± 20.70 ms (*N* = 10, Figure 4E). Thus, the time to onset is shorter [*t*_(243.287)_ = 3.621, *p* < 0.001] with a smaller variance [Levene's test: *F*_(61.36)_, *p* < 0.001] for strong stimulation indicating that release from strong synchronous PC inhibition leads to more precisely timed CN firing compared to weak PC-mediated inhibition.

### Rebound firing in cerebellar nuclei neurons follows timed offset of purkinje cells

In most CNs (10 out of 13) optogenetically-induced inhibition was followed by a rebound wave of excitation, which lasted up to tens of milliseconds. We did not observe a relation with the occurrence or the strength of rebound firing and cell physiological parameters such as membrane resistance, nor with the recording location. Rebound excitation was often biphasic with an initial excitation followed by inhibition and a second excitation (Figure 4D). The timing of the peak excitation as well as the first inhibition and second excitation [as determined by convolving the spike train with a Gaussian of width σ = 1 ms, see Materials and methods, (Hoebeek et al., 2010)] after either 500 or 1000 ms of PC stimulation were not significantly different [500 vs. 1000 ms; first peak: 32.2 ± 17.0 vs. 45.7 ± 30.8, *t*_(15)_ = 0.95, *p* = 0.36; inhibition: 50.4 ± 11.9 vs. 62.6 ± 20.9, *t*_(12)_ = 1.13, *p* = 0.28; second peak: 85.8 ± 28.6 vs. 91.8 ± 20.4, *t*_(10)_ = 0.38, *p* = 0.71]. The average firing rate over the period after the stimulus (100 and 200 ms, for both 500 and 1000 ms) was significantly higher than the pre-stimulus firing rate in all comparisons [*F*_(1, 35)_ = 14.69, *p* < 0.001, and *F*_(2, 41)_ = 13.82, *p* < 0.001 for 500 and 1000 ms stimulation, respectively; *post-hoc* all *p* < 0.001] (Figures 4F–H). Comparing different stimulus strengths revealed that five out of eight cells showed a significantly stronger inhibitory response during the stimulus when the stimulus strength was increased (power from 8.59 ± 8.55% to 58.89 ± 25.07%; ANOVA; *p* < 0.001, Figures 4F,G). Similarly, five out of eight cells showed a significantly stronger rebound after stronger light stimulation (ANOVA; *p* < 0.001) (Figure 4G). Thus, the strength of this rebound was also related to the strength and duration of the light stimulus. In general it was the case that cells showing strong inhibition also showed rebound firing (Figure 4H).

### Evoked movements follow termination of synchronously activated purkinje cells in awake mice

**Figure 5:**
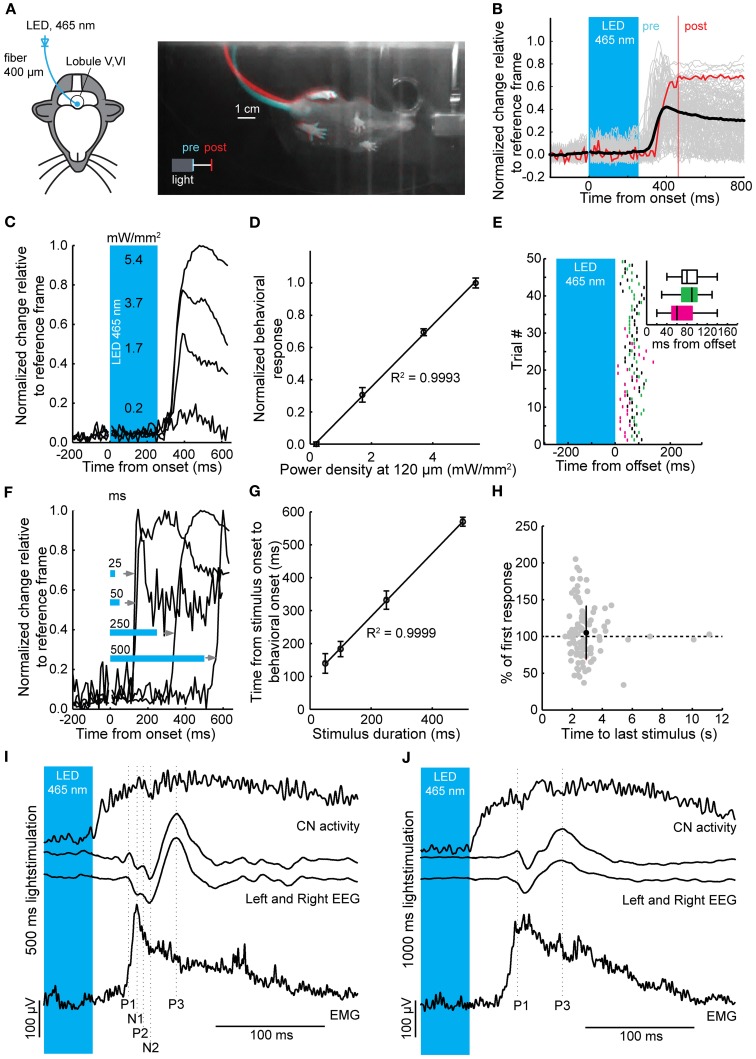
**Timed motor responses in awake mice during optogenetic activation of PCs. (A)** For the behavioral assay head-fixed mice were placed on a transparent disc that could freely rotate. The optic fiber was placed on the brain surface of lobules V and VI (left) for optogenetic stimulation. Light was delivered to the brain via a LED coupled to the optic fiber. Right: Bottom view of a mouse responding to optogenetic activation of PCs (250 ms, ~5 mW/mm^2^) with a twitch of its tail and hind legs after stimulus offset. Camera frames were acquired at 100 Hz. Differences between two frames at the stimulus offset (“pre,” cyan) and 200 ms post-offset (“post,” red) show relative position change between the two time points. **(B)** Individual behavioral responses (gray traces), response corresponding to twitch shown in **(A)** [red trace, one frame chosen at offset (pre), and one 200 ms post-offset (post)] and mean behavioral response (black trace) following a 250 ms light stimulus. **(C)** Behavioral responses were graded with increases in light intensity. Estimated power densities are shown at a depth of the PC monolayer (~120 μm). **(D)** Normalized behavioral response plotted vs. power density showing a linear correlation (R^2^ = 0.9993, slope = 0.19). **(E)** Raster plot showing individual behavioral onsets relative to the stimulus offset (time = 0). Inset: box plots (three mice indicated by different colors) of behavioral onsets relative to the stimulus offset (whiskers indicate distance from 25 to 75% interquartile ranges to furthest observations, center mark represents the median). **(F)** The onset of behavior shifted with an increase in stimulus duration, such that the relative delay to a behavioral response onset relative to stimulus offset was maintained. Note that behavioral responses can be elicited by stimuli with durations of 25 ms. **(G)** Time from stimulus onset to behavioral onset plotted against stimulus duration followed a linear relationship (R^2^ = 0.9999 and slope = 0.96) demonstrating that the onset of behavioral responses shifts relative to the stimulus duration. **(H)** The interstimulus interval did not have an effect on strength of the behavioral response (*r* = −0.071, *p* = 0.49). **(I)** and **(J)** Simultaneous recordings of CNs, bihemispheric EEG, and EMG to 500 ms **(I)** and 1000 ms **(J)** light stimulation of PCs in anesthetized mice. For clarity, the stimulation period has been truncated and only the last 45 ms of the stimulus is shown in the blue box. Vertical scale bars apply to both EEG traces and EMG traces in **(I)** and **(J)**. Top panels, average Gaussian-convoluted spike train of all CNs. Middle panels, left and right EEG. Bottom panels, rectified, differentiated and again rectified EMG responses. The vertical dotted lines indicate the location of the positive (P1 to P3) and negative (N1 to N2) deflections in the EEG signals. Note that the onset of the EMG response occurs before the first response peak in the EEG, while the EMG signal itself is preceded by CN activity.

To directly investigate the impact of light stimulation of PCs on movements, we optogenetically stimulated PCs over lobules V and VI in awake mice (Figure 5A) (Stark et al., 2012). These cerebellar lobules have been reported to show zonal proximal limb and tail representations in cats and rodents (Provini et al., 1968; Robertson, 1984; Buisseret-Delmas and Angaut, 1993; Jorntell et al., 2000; Ekerot and Jorntell, 2001). Mice were placed in a dark environment on a freely rotating transparent disc to allow recording of behavior from underneath with an infrared camera (Figure 5A), while we stimulated an estimated 400 PCs (see Materials and Methods) with flashes of blue light. Stimulations in resting mice resulted in stereotypical twitches of tail and proximal limbs (Figures 5B–F). Robust behavioral responses could be elicited by stimuli ranging from 25 to 500 ms (Figure 5F). In line with PC and CN responses, the behavioral response was graded and linearly related to the power density of the light stimulus (R^2^ = 1.00) (Figure 5D), while the onsets of the muscle contractions were strongly related to the offset of the stimulus (R^2^ = 1.00) (Figures 5E–H). The behavioral response was delayed with respect to the end of the stimulus by an average of 81.5 ± 27.9 ms (129 trials, *N* = 3 mice; 68.7 ± 36.0 ms, 85.1 ± 24.8 ms, 86.3 ± 22.2 ms for individual mice) (Figure 5E). The strength of the response did not diminish or enhance with repeated activation for the intervals used (*r* = −0.07, *p* = 0.49; mean response: 104.9 ± 36.5% of first response at 2.9 ± 1.4 s) (Figure 5E).

### Muscle contractions resulting from synchronously activated purkinje cells are not mediated by cerebral cortex

To examine whether the cerebral cortex was required to initiate movements following optogenetic stimulation of the cerebellar cortex we recorded electroencephalograms (EEGs) from primary motor cortex and electromyograms (EMGs) from the musculus biceps femoris of the hind limb in anesthetized mice, while stimulating PCs and recording CN activity in the medial cerebellar nucleus (*N* = 14) (Figures 2A, 5I,J). Stimulation-offset triggered averages of the cortical EEG showed a stereotypic EEG waveform consisting of a sequence of peaks and troughs (P1, N1, P2, N2, and P3 subsequently, *N* = 14) (Figures 5I,J). The timing between the left and right EEG for these components was identical for 500 and 1000 ms light stimulations (Table 1). Apart from yielding a robust response in the cortical EEG, stimulations of 500 to 1000 ms duration resulted in stereotypical twitch responses in the tail and proximal limbs of anesthetized mice. The onset of muscle twitches was related to the termination of the light stimulus, with the maximal rectified EMG response at 48.0 ± 10.3 ms after stimulus offset [41.2 ± 2.2 ms and 52.0 ± 11.2 ms after 500 and 1000 ms stimulation, respectively, *t*_(18)_ = 3.07, *p* = 0.007; *N* = 7, and *N* = 12] (Figures 5I,J). Instead, the onset of the EMG response occurred earlier at 36.18 ± 11.05 ms [30.52 ± 7.21 ms and 39.48 ± 11.79 ms after 500 and 1000 ms stimulation, respectively *t*_(18)_ = 2.04, *p* = 0.055; *N* = 7, and *N* = 12], which places it at similar times as the first input to the cerebral cortex (Meeren et al., 1998). Thus, the movements evoked by optogenetic stimulation of the cerebellum were likely initiated via a direct pathway (e.g., red nucleus and/or lateral reticular formation) and not through projections to the cerebral cortex.

**Table 1:**
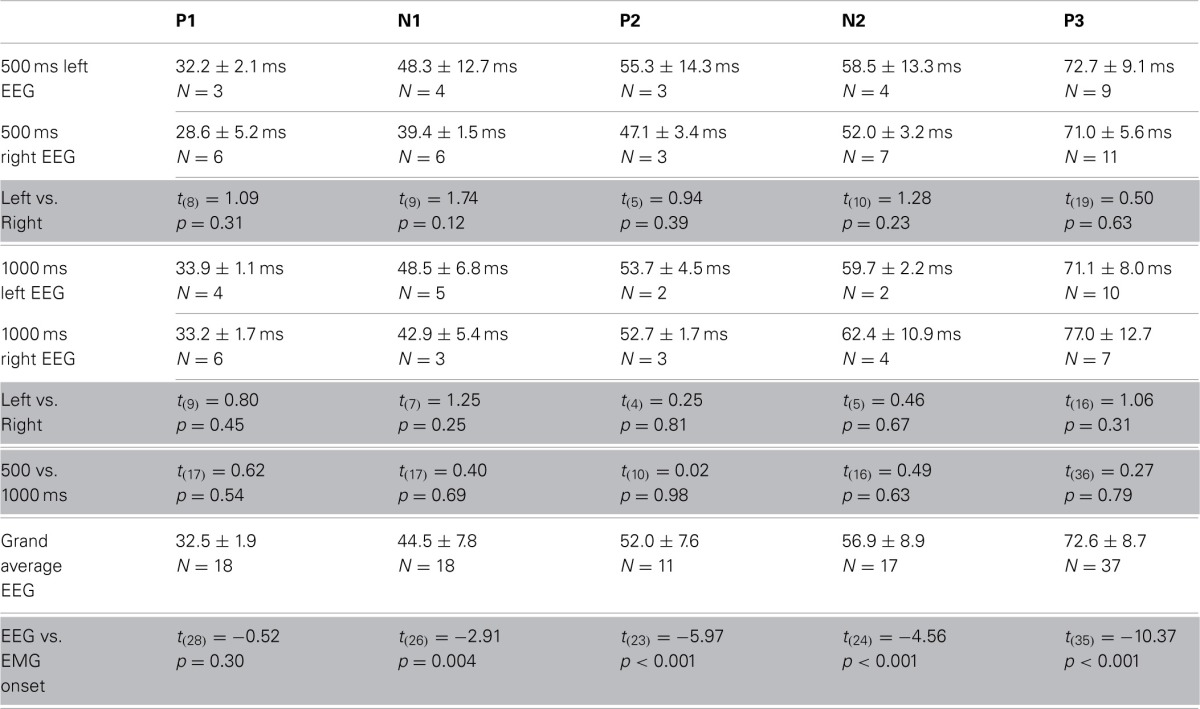
**Timing of EEG and EMG components**.

Timing of motor cortex EEG relative to optogenetic activation of PCs. Each column lists the average delay from stimulus offset to the indicated response. Each row lists a different condition or statistical test. N lists number of sets of ten traces analyzed.

### Modulation of the olivo-cerebellar feedback loop by purkinje cells

**Figure 6:**
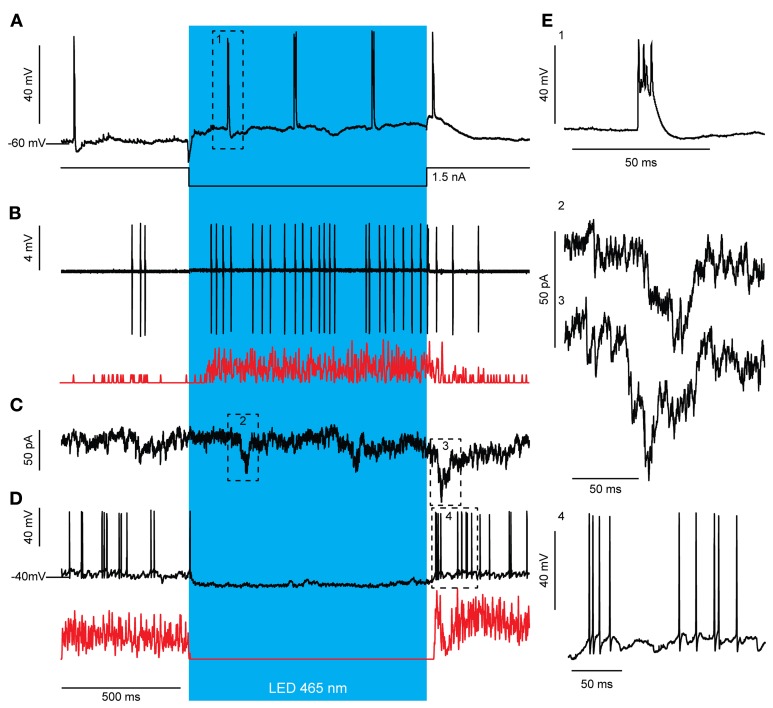
**Optogenetic stimulation of PCs elicits an increase in CS activity, which is most likely a network effect. (A)** Light stimulation (blue bar) evokes an increase in CS activity even when the SS increase is prevented by intracellular current injection via the patch electrode. Additionally, a CS was observed after stimulus offset. Notice the depolarized membrane potentials after stimulus offset indicating slow inactivation of the ChR2 (H134R) channel (arrows). **(B)** MLI activity is not directly increased in response to PC stimulation but after >50 ms delay. This activation is likely due to the recorded CS increase **(A)** that leads to MLI activation through glutamate spillover. The increase in MLI firing frequency outlasted the light stimulus [see also Gaussian-convoluted trace in red; Putative MLIs, *N* = 3; baseline firing rate 50 ms before light stimulation: 1.69 ± 9.54, firing rate <50 ms after strong light stimulation: 1.93 ± 8.97, *t*_(123)_ = −0.421, *p* = 0.675] similar to what we see for CS activity **(A)**. **(C)** A voltage and subsequent current clamp recording **(D)** of a single representative CN during light stimulation of PCs. **(C)** Voltage clamp recordings of CNs reveal several large, summating EPSCs present during and directly after the light stimulus (arrows), which may be evoked by the increased climbing fiber activity **(A)**. **(D)** In current clamp, the inhibition from firing during PC stimulation (blue bar) and the biphasic rebound activity after the inhibition is visible. Note, the timing of the CS activity **(A**, **C)** of PCs after offset of the light stimulus precedes the break in the CN rebound (**D**, see also Gaussian-convoluted trace in red). **(E)** Example complex spike from the trace in **(A)** (1), example EPSCs from the trace in **(C)** (2 and 3), and magnification of rebound firing in **(D)** (4).

**Figure 7:**
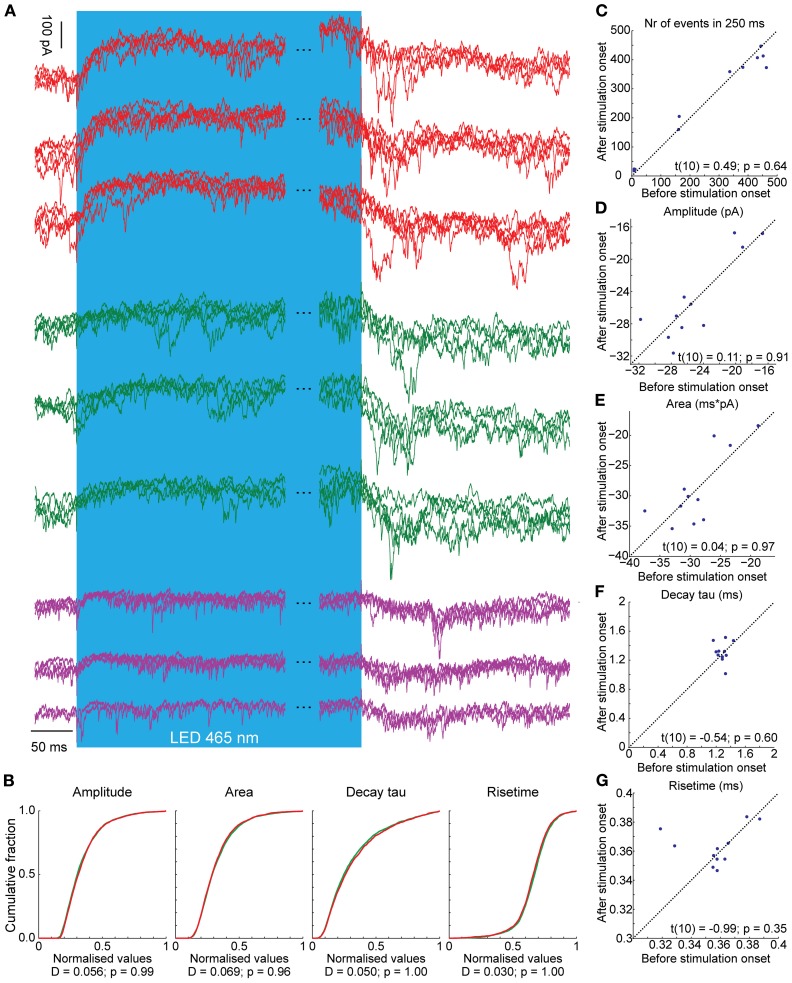
**Voltage clamp recordings of three CNs during light stimulation of PCs (blue bar, the time during the stimulus is not completely shown; notice the break between the blue bars).** Three representative CNs are shown. Per neuron we show three overlays of each four traces (so, a total of 12 traces per cell). **(A)** CNs react with an outward current in response to the light stimulus. In addition, there are several large, summating EPSCs present during and directly after the light stimulus, probably induced by climbing fiber activity. **(B)** There were no differences in the distributions of various EPSC kinetics before (green) and after (red) stimulation onset. Also, the **(C)** number of events, **(D)** amplitude of the excitatory postsynaptic potentials, **(E)** the area, **(F)** the decay and **(G)** rise time do not differ before and after stimulation onset, suggesting that the mossy and climbing fiber inputs share similar kinetics. The significance of pairwise comparisons is listed for each panel separately.

Optogenetic stimulation of PCs elicited robust SS activity (Figure 3). This, in theory should lead to inhibition of GABAergic CNs that project to the IO and a resulting disinhibition of olivary neurons to cause an increase of CS activity. This prediction indeed holds. During light activation for 1000 ms the average CS rate (*N* = 7) increased significantly from a baseline of 0.73 ± 0.38 Hz to 1.54 ± 0.89 Hz and 1.84 ± 0.45 with a low and high stimulus strength, respectively [baseline vs. weak *t*_(12)_ = −2.194, *p* = 0.049; weak vs. strong *t*_(6.002)_ = −2.811, *p* = 0.031; baseline vs. strong *t*_(6)_ = −2.841, *p* = 0.030]. The observed increase in CS activity, which occurs consistently throughout trials, might in principle result from single cell connections in the olivocerebellar loop, but it may be facilitated through more extensive network properties in that multiple PCs of the same sagittal zone converge onto individual CNs (De Zeeuw et al., 2011). When the membrane depolarization of a single PC during light stimulation *in vivo* was prevented by hyperpolarizing current injections, the SS frequency of that particular cell did not increase, whereas its CS rate increased persistently during and directly after the light stimulus that was applied to multiple PCs within a zone (Figure 6A). This indicates that the network properties of an ensemble of PCs are sufficient to induce an increase in CS activity, even when the SS activity of the recorded PC is suppressed. If the CS activity of a particular zone is enhanced following optogenetic stimulation of PCs through the network properties of the olivocerebellar loop, one expects that the activity of MLIs, which receive climbing fiber input through spillover (Jorntell and Ekerot, 2003; Szapiro and Barbour, 2007), will also be increased once the CS increase occurs, but not earlier than that. Indeed, MLIs responded to a 1000 ms light stimulation with a significant increase in firing frequency from 11.61 ± 2.43 Hz to 28.89 ± 4.32 Hz [*t*_(4)_ = −3.476, *p* = 0.025; *N* = 3], but this increase was delayed for more than 50 ms relative to the onset of the light stimulus reflecting elapsed time prior to disinhibition of the IO by the light stimulus (Figure 6B). We observed several large postsynaptic events in voltage clamp recordings of CNs both during and after light stimulation, which probably reflect climbing fiber collateral input (Figure 7). In addition, activity in the climbing fibers probably also facilitated late CN rebound via their collaterals (Figures 6C,D, 7), because during voltage clamp recordings of CNs we observed putative climbing fiber-mediated excitatory postsynaptic currents (EPSCs) within 50 to 100 ms after termination of the light stimulation coinciding with the moments when CSs occur in PCs (see also Figure 7). Indeed in PCs, a robust but loosely timed CS was observed after stimulus offset. For 1000 ms excitation of PCs this CS had an average latency of 73.11 ± 32.73 ms (*N* = 6), whereas for 500 ms excitation this latency (82.13 ± 49.43 ms) was slightly, but not significantly longer [*t*_(275.53)_ = −1.904, *p* = 0.058]. Taken together, these observations indicate that light-driven activation of PCs is effective in disinhibiting the IO and that the timing of the CS activity of PCs and that of the activity in the presumptive climbing fiber collaterals after offset of the light stimulus both correlate well with the temporal characteristics of the rebound in CNs.

## Discussion

Over the past years various studies have shown that synchronous activation of PC ensembles is essential for the transfer of behaviorally relevant information from the cerebellar cortex to the cerebellar nuclei (Bell and Grimm, 1969; Sjolund et al., 1977; Sasaki et al., 1989; De Zeeuw et al., 1993, 1997a, 2011; Welsh et al., 1995; Levin et al., 2006; Walter et al., 2006; Heck et al., 2007; Catz et al., 2008; de Solages et al., 2008; Van Der Giessen et al., 2008; Ozden et al., 2009; Schultz et al., 2009; Wise et al., 2010; Person and Raman, 2012a,b). Yet, technical limitations have hampered intracellular *in vivo* whole cell recordings of CNs and selective PC stimulation. In the present study, we used the Ai32 (ChR2(H134R)-eYFP) transgenic mouse and a L7-Cre driver line to allow for selective and temporally well controlled activation of PCs and combined this with *in vivo* whole cell recordings to examine the effect of well-timed PC activation on CNs and the olivo-cerebellar network. Using whole cell *in vivo* recordings of PCs and CNs we have shown that timed light onset evokes synchronized activation of PCs. This is supported by the findings that evoked inhibitory events in CNs were visible in response to light stimulation and that these inhibitory potentials summated well, demonstrating that a CN receives multiple synchronized events. With increased light intensity, shorter latency responses with a reduced variation in the onset time of PCs were observed, suggesting that with a reduction in variability more synchronization occurs. To the best of our knowledge, this is the first study showing how the olivo-cerebellar network responds to synchronized activation and subsequent deactivation of PCs and how such synchronization may generate timed motor responses.

### Rebound firing evoked by synchronous PC disinhibition

As suggested earlier, we find that timed release from PC inhibition leads to a signature rebound response in CNs (Aizenman and Linden, 1999; Nelson et al., 2003; Hoebeek et al., 2010; De Zeeuw et al., 2011). We also show that by increasing the strength of the preceding PC light-stimulation, the onset of rebound activity becomes more precisely timed. This matches our previous findings in which olivary stimulation was more effective in evoking rebound in CNs than focal electrical, cortical stimulation (Hoebeek et al., 2010). Complementing and extending previous studies (Jahnsen, 1986; Aizenman and Linden, 1999; Molineux et al., 2006, 2008; Pugh and Raman, 2006; Alvina et al., 2008; Steuber et al., 2011) we demonstrate that rebounds can be observed even when completely silenced prior to the rebound. This can be explained by a massive distributed input from the orchestrated activation of PCs by our light stimulus compared to a point-source current injection at the soma (Gauck et al., 2001). Therefore, subtle changes in the timing of PC activity could already lead to pivotal CN firing adjustments that could influence not only behavior but also CN plasticity by timed coding (Pugh and Raman, 2006). Indeed, we show that even weak activation of an ensemble of PCs is sufficient to evoke rebounds *in vivo*.

### Timed purkinje cell inactivation evokes muscle contractions

The cerebellum may modulate ongoing movement and specific reflexes in part through synchrony of PC CS firing, likely causing larger and more sudden changes in motor output the more synchronized CSs are involved (De Zeeuw et al., 2011). Key to this hypothesis is the synchrony and magnitude with which changes in a PC population's ongoing SS activity occur, as a CS occurrence has a profound effect on SS activity and the SS coding is assumed to shape the continuous output from the cerebellum that is required for ongoing motor control. We were able to show that light-driven SS modulation in PC ensembles is able to control rebound activity in CNs and subsequently regulate the onset of motor behavior via cessation of PC stimulation. To determine how synchronous activation of the cerebellum could possibly influence timed motor responses, we recorded simultaneously the EEG of the motor cortex and the EMG of the biceps femoris of anesthetized mice. In an extra set of experiments we monitored evoked movements in awake mice. We found that EEG responses and muscle twitches are timed to the offset of the light stimulus rather than the onset. Furthermore, our data show that the CN rebound rather than PC activity is related to the onset of synchronous activity in the motor cortex (Fujikado and Noda, 1987; Noda and Fujikado, 1987; Godschalk et al., 1994). Despite the relatively fast first response in neocortical EEG, it is not possible that all behavioral output generated in our experiments is mediated and initiated via the motor cortex, since the onset of the EMG response occurred at similar times as the first response in the EEG, which reflects thalamic input to the neocortex (Meeren et al., 1998). Therefore, we propose that at least the initial part of the behavioral output, as measured with EMG and in our awake behavioral assay, is mediated via other routes than projections through thalamus and motor cortex. A more direct route probably relies on brainstem nuclei such as the red nucleus and/or lateral reticular formation (Teune et al., 2000). Altogether, we demonstrate that light-driven activation of PC ensembles is able to regulate the onset of motor behavior via graded control of rebound activity in CNs.

### Activation of PC ensembles modulates the olivo-cerebellar feedback loop

PCs responded to graded light activation with a graded increase in the firing rate of SS and CS. We show here that the increase of CS rate was not a direct effect of the channelrhodopsin stimulation upon the cell, but rather a result of the activation of the olivo-cerebellar feedback loop. An increase in SS rate depresses the CN, including the inhibitory projections to the IO (De Zeeuw et al., 1988; Angaut and Sotelo, 1989; Ruigrok and Voogd, 1990; Fredette and Mugnaini, 1991). Such disinhibition of the IO may increase the activity and rhythmicity of the climbing fibers (Stratton and Lorden, 1991; Lang et al., 1996; Bengtsson et al., 2004). CS rate increased independent of membrane voltage as shown by experiments in which single PCs were hyperpolarized with current injections, supporting the idea that the persisting increase of CSs was caused by reverberation in the olivo-cerebellar loop. The fact that rebound firing was biphasic due to synchronous CS firing in PCs further underscores the idea that PCs-CNs-IO neurons form a closed feedback loop (Lang et al., 1996; Marshall and Born, 2007). Thus, by modulating their own firing, PCs may be able to influence climbing fiber dependent plasticity and conditioning (Rasmussen et al., 2008).

The olivo-cerebellar loop and its impact on the cerebellar cortical network may also explain in part why PC-mediated inhibition could evoke first a deep hyperpolarization in CNs and subsequently, after a short few millivolt recovery, some spike activity although the light stimulus was maintained (Figure 4D). Possibly, IO disinhibition by CN inactivation could provide enough excitatory input from climbing fiber collaterals to CNs to drive spike firing during PC-mediated inhibition (Van der Want et al., 1989; De Zeeuw et al., 1997b; Ruigrok and Voogd, 2000). Indeed, in voltage clamp we often observed EPSCs in CNs after several ms of PC inhibition (Figures 6, 7). An additional explanation may be found in the fact that PC to CN synapses show profound short-term depression (Telgkamp and Raman, 2002; Pedroarena and Schwarz, 2003; Luthman et al., 2011), which can limit the synaptic current during strong PC activation. Such short-term depression was also observed during activation of PCs while voltage-clamping CNs (Figures 4, 6, 7). Finally, hyperpolarization-activated depolarizing currents, which were observed before in CNs (Aizenman and Linden, 1999; Molineux et al., 2006; Engbers et al., 2011), can limit the extent of the hyperpolarization induced by synaptic inputs.

Although we did see an apparent increase in the occurrence of high amplitude EPSCs during and directly after the light stimulation, overall the distributions and averages of all postsynaptic excitatory events did not change before and after stimulus onset. This indicates that climbing fiber collateral-mediated EPSCs do not have different kinetics from mossy fiber collateral-mediated EPSCs, which are expected to make up the majority of excitatory inputs to CNs. Even though our data seems to indicate a functional equivalence of mossy and climbing fiber collaterals, more experiments are needed to directly address this issue.

We conclude that temporally appropriately configured activity and silencing of ensembles of PCs will allow graded control of rebound activity in CNs and thereby motor activity, and that this control may be supported by reverberating activity in the modular olivo-cerebellar loops.

## Materials and methods

All procedures adhered to the European guidelines for the care and use of laboratory animals (Council Directive 86/6009/EEC). Protocols were also approved by the animal committee of the Royal Netherlands Academy of Arts and Sciences (DEC-KNAW). L7-cre mice were crossed with ChR2(H134R)-eYFP mice to obtain L7-ChR2(H134R)-eYFP animals which express the channelrhodopsin-2 H134R variant (Berndt et al., 2011; Madisen et al., 2012) under control of the L7 promoter (Oberdick et al., 1990). Mice (*N* = 17) were prepared for the experiment by placing three EEG connectors and a pedestal on the skull under isoflurane anesthesia (1.5% in 0.5 l/min O2 and 0.2 l/min air). The skin on top of the head was shaved and cut sagittaly to expose the bone. The bone was then quickly etched with phosphoric acid gel (37.5%) and washed with saline. Three <2 mm diameter holes for the EEG electrodes were drilled over the motor cortices (1.5 mm frontal and 2.0 mm lateral from bregma) and over the parietal cortex. EEG electrodes were made from silver wires soldered to IC connectors. The silver wires were bent at the end as to protect the dura from puncturing and carefully inserted into the holes. Primer and adhesive were applied according to manufacturer's specification (Kerr, Orange, California). A pedestal, consisting of two M1.4 nuts soldered together, was attached to the skull with dental acrylic (flowline; Heraeus Kulzer, Hanau, Germany). Care was taken to incorporate the EEG electrodes in the pedestal and to come to a solid block on top of the mouse's skull. The skin was then sutured to obtain a nice connection to the pedestal. Animals received analgesia in the form of Metacam (AUV, 2 mg/kg) and were allowed to recover for at least 1 day.

### In vivo patch clamp and extracellular recordings

On the day of the experiment animals received an initial i.p. injection of ketamine/ xylazine (75 and 12 mg/kg) and supplemented when needed. Animals were kept at 37°C body temperature via a feedback controlled heating pad. The mouse was fixed in the setup via the pedestal, the cerebellar cortex was revealed by drilling a large hole covering most of the occipital bone and the dura mater was removed. EMG electrodes consisted of a syringe needle (25G) connected to the amplifier. EMG electrodes were inserted in the biceps femoris of the hind leg. EEG leads were connected to the IC connectors on the skull of the mouse on one end and to a simple amplifier, together with the EMG electrode lead (adapted MEA60, Multichannel systems, Reutlingen, Germany). Whole-cell recordings of CNs were made using borosilicate glass electrodes (Harvard Apparatus, Holliston, Massachusetts) with 1- to 2-μ m tips and 8 to 12 MΩ, filled with internal solution (in mM: 10 KOH, 3.48 MgCl2, 4 NaCl, 129 K-Gluconate, 10 hepes, 17.5 glucose 4 Na2ATP, and 0.4 Na3GTP), amplified with a Multiclamp 700B amplifier (Axon Instruments, Molecular Devices, Sunnyvale, California), and digitized at 50 KHz with a Digidata 1440 (Axon Instruments, Molecular Devices, Sunnyvale, California, United States).

### In vivo voltage clamp recordings of CNs

CNs were patched as described above. Voltage clamp recordings were obtained in a subset of cells with sufficiently low access resistance (<50 MΩ). Neurons were clamped at voltages between −60 and −75 mV, which was sufficient in all cases to prevent voltage escape inducing spikes. After voltage clamp recordings were completed, the cell was recorded in current clamp following the exact same stimulation parameters. From one cell we normally could obtain recordings from both 500 and 1000 ms stimulation durations.

### Light stimulation for in vivo patch clamp and extracellular recordings

For strong, timed stimulation of channelrhodopsins, we developed a LED driver capable of driving three LEDs at a maximum of 5 watts of power per LED. Light intensity was set for the latter with a ten-turn dial for LED-light stimulation. Three LED lights (465 nm, 60 lm, LZ1-B200, LED Engin, San Jose, California), positioned around the cerebellum of the mouse, were used to illuminate the whole cerebellum (Figure 2A). This stimulus was powerful enough to activate PCs on every trial (Figures 3, 4).

### Data analysis of in vivo patch clamp and extracellular recordings

Latencies to first spike for PCs were calculated as the time difference between the first spike and the onset of the stimulus, while for CNs the offset of the stimulus was used.

Firing rate increases and decreases were calculated of the period of 999 or 499 ms during the stimulus (for 1000 and 500 ms stimulation lengths resp.) and an equal time before the stimulus. Gaussian convolution of spike trains was done as described previously (Hoebeek et al., 2010). In short, each spike time was convolved with a Gaussian distribution (kernel) with peak 1 and width (σ) of 1–20 ms. Patch clamp data was analyzed in Clampfit (Axon Instruments, Molecular Devices, Sunnyvale, California, United States) to detect spikes and to measure membrane potential and membrane currents. EEG, EMG, and Gaussian-convolved traces were analyzed in Matlab (R2010b, Mathworks, Natick, Massachusetts, United States). Raw EMG recordings were low-pass filtered up to 500 Hz, then rectified, differentiated and again rectified. This resulted in a clear signal at the time at which motor endplate activity could be discerned as high frequency activity in the raw signal.

### Behavioral assay of purkinje cell activation

Mice were head-fixed and placed on a freely rotating transparent disc before light stimulation experiments commenced. The disc was secured on a ball bearing, to ensure that forces exerted by the animal would not compromise head fixation and mice could move at will. A blue LED (465 nm, see above) was coupled into a 400 μm multimode optical fiber (Thorlabs, Newton, New Jersey), which was placed at the border of the anterior vermis regions lobule V and VI through a small (0.5–1 mm) drilled hole. The hole was covered with Kwik-Sil (World Precision Instruments, Sarasota, Florida) and the fiber was secured with dental cement. (Super-Bond, Generinter, France). Our custom-made LED driver was used to apply linearly increasing amounts of light intensity. We estimated the number of activated PCs by first calculating surface area at the bottom of the cone of light emitted from the fiber:

$$A={\left\{\left(\frac{r_{\text{fiber}}}{\tan\left(\sin^{-1}\frac{NA_{\text{fiber}}}{NA_{\text{brain}}}\right)}+d\right)*\tan\left(\sin^{-1}\frac{NA_{\text{fiber}}}{NA_{\text{brain}}}\right)\right\}}^{2}*\pi$$

Where *r*_fiber_ is the radius of the fiber, *NA*_fiber_, and *NA*_brain_ are the numerical apertures of the fiber and brain tissue (0.37 and 1.35 resp.) and *d* is the depth in μm; *A* is defined in μm^2^. For the current experiments this means a radius of 234.2 μm (120 μm depth, 0.172 mm^2^). Light is spread over this area and is attenuated by scattering and absorption by brain tissue following the rule (Yizhar et al., 2011):

$$P=100\%*e\frac{-2.556*d}{1000}$$

Where *P* is the resulting power at depth *d* in percent of the original power from the fiber tip. For the current set of experiments we obtained 73.6% power of the original 1.325 mW. This was spread over a surface of 0.17 mm^2^, resulting in 5.66 mW/mm^2^, which should be sufficient for reliable channelrhodopsin activation (Berndt et al., 2011). Harvey and Napper (1991) estimate the density of PCs in the rat cerebellum to be 936 PCs/mm^2^, which would correspond to 161 PCs in the illuminated area. A theoretical maximum is given by the optimal hexagonal packing of circles within the illuminated area:

$$\eta =\frac{1}{6}*\pi *\sqrt{3}\approx 0.9069$$

With η representing the packing density. Assuming a PC soma diameter of 22 μm, this gives a theoretical maximum of 411.09 stimulated PCs. Therefore, we estimate that with the current light fiber we stimulate 150–400 PCs.

Behavior was recorded at 100 Hz with an infrared camera. In order to detect movements caused by light-driven activation of PC ensembles, a custom-written twitch detection algorithm was used to extract twitch responses from a sequence of camera frames. First, the frame coinciding with the onset of the TTL pulse to the LED stimulation box was selected as a reference frame. This frame was de-noised using a median filter of 5-by-5 pixels. Then, 20 frames before and 80 frames after were analyzed by the algorithm (thus spanning a total length of 1010 ms, including the reference frame). The reference frame was subtracted from all frames in the analyzed sequence:

$$y_{n}=\|x_{n}-x_{ref}\|$$

where *y*_*n*_ is the resultant image at the *n*^*th*^ position of the processed image sequence, *x*_*n*_ the original image and *x*_*ref*_ the reference image. The resultant frames were then flattened to two separate 1-dimensional vectors representing both the vertically and horizontally summed difference values:

$$\begin{array}{l} {\hat{v}}_{n}{\left(q\right)}_{vt}=\sum_{k=1}^{m}y_{n}(q,k)\\ {\hat{v}}_{n}{\left(q\right)}_{hz}=\sum_{k=1}^{p}y_{n}(q,k) \end{array}$$

where *v*_*vt*_ and *v*_*hz*_ are the summed difference values taken vertically and horizontally, respectively; *m* and *p* are the width and height of the image (in pixels), respectively; and *q* is the position of the value in vector *v*, corresponding with either an image line or column. For the first 20 vectors in both dimensions, the standard deviation in values per position was determined. Based on these values, a weighting vector was constructed for both the vertical and horizontal dimension vectors:

$${\hat{w}}_{\dim}={\hat{\sigma }}_{\dim}^{-1}$$

where *w* is the weighting vector, *dim* denotes the dimension (vertical or horizontal) and σ is the vector containing standard deviations. The inner product of the weighting vectors and the summed difference value vectors were then used to get one mean change trace:

$$t_{n}=\frac{\left({\hat{w}}_{vt}\cdot {\hat{v}}_{n,vt})+({\hat{w}}_{hz}\cdot {\hat{v}}_{n,hz}\right)}{2}$$

where *t*_*n*_ is the trace value at index *n*. The mean and variance for the first 20 values of *t* were then determined. A deviation of more than four standard deviations from the mean as based on the first 20 values of *t* was counted as a twitch.

### Conflict of interest statement

The authors declare that the research was conducted in the absence of any commercial or financial relationships that could be construed as a potential conflict of interest.
